# A Spiritual Self-Care Mobile App (Skylight) for Mental Health, Sleep, and Spiritual Well-Being Among Generation Z and Young Millennials: Cross-Sectional Survey

**DOI:** 10.2196/50239

**Published:** 2023-09-27

**Authors:** Susanna Y Park, Jennifer Huberty, Jacqlyn Yourell, Kelsey L McAlister, Clare C Beatty

**Affiliations:** 1 Skylight, Radiant Foundation Salt Lake City, UT United States; 2 Fit Minded, Inc Phoenix, AZ United States

**Keywords:** Gen Z, millennial, spirituality, spiritual self-care, mental health, sleep, mobile health, mHealth, digital health, spiritual, self-care, app, apps, mental wellness, mental well-being, young adult, young adults, engagement, perception, perceptions

## Abstract

**Background:**

Generation Z (Gen Z) and young millennials (GenZennials) (ages 18-35 years) are unique in that they either have no memory of or were born shortly after the internet “explosion.” They are constantly on the internet, face significant challenges with their mental health and sleep, and are frequent users of digital wellness apps. GenZennials also uniquely identify with and practice spirituality, which has been linked to better mental health and sleep in adult populations. Research has not examined digital approaches to spiritual self-care and its relationship to mental health and sleep in GenZennials.

**Objective:**

The purpose of this study was to describe a sample of adult GenZennials who use a spiritual self-care app (ie, Skylight), describe how users engage with and perceive the app, and assess the relationship between frequency of using the app with mental health, sleep, and spiritual well-being.

**Methods:**

Participants were 475 adult Gen Z (ages 18-28 years) and young millennial (ages 29-35 years) Skylight app users who responded to an anonymous survey on the web. The survey asked about demographics, spiritual self-care and practice, and user engagement and perceptions of the app. Outcome measures included 4 validated surveys for mental health (ie, depression, anxiety, and stress) and sleep disturbance, and one validated survey on spiritual well-being. Mean scores were calculated for all measures, and linear regressions were conducted to examine the relationship between the frequency of app use and mental health, sleep, and spiritual well-being outcomes.

**Results:**

Participants were predominantly White (324/475, 68.2%) and female (255/475, 53.7%), and approximately half Gen Z (260/475, 54.5%) and half young millennials (215/475, 45.3%). Most users engaged in spiritual self-care (399/475, 84%) and said it was important or very important to them (437/475, 92%). Users downloaded the app for spiritual well-being (130/475, 30%) and overall health (125/475, 26.3%). Users had normal, average depressive symptoms (6.9/21), borderline abnormal anxiety levels (7.7/21), slightly elevated stress (6.7/16), and nonclinically significant sleep disturbance (5.3/28). Frequency of app use was significantly associated with lower anxiety (Moderate use: β=–2.01; *P*=.02; high use: β=–2.58; *P*<.001). There were no significant relationships between the frequency of app use and mental health, sleep, and spiritual well-being outcomes except for the personal domain of spiritual well-being.

**Conclusions:**

This is the first study to describe a sample of adult GenZennials who use a spiritual self-care app and examine how the frequency of app use is related to their mental health, sleep, and spiritual well-being. Spiritual self-care apps like Skylight may be useful in addressing anxiety among GenZennials and be a resource to spiritually connect to their personal spiritual well-being. Future research is needed to determine how a spiritual self-care app may benefit mental health, sleep, and spiritual well-being in adult GenZennials.

## Introduction

### Background

More than half of the US population (166 million) are now Generation Z (Gen Z), millennials, or younger [1]. Gen Z includes individuals born between 1995 and 2012 and millennials include those born between 1981 and 1996 [2]. These generations were born into an age of the internet and wide technology access. Younger millennials were born soon after the internet “explosion,” and Gen Z followed closely behind being born into a world where they would never know what it is like to live without the internet and technology [3]. In a survey of 1052 US adults by the Pew Research Center, almost half of 18- to 29-year-olds and 42% of 30- to 49-year-olds reported being on the web “constantly” [4]. Gen Z and young millennials, referred to as GenZennials, are unique in that they are “digital natives,” do not understand what it truly means to “log off” [5], and thus are “always on [3].” This study focuses on adult GenZennials (ages 18-35 years).

The state of being “always on” for both generations has public health implications, such as poorer psychological and physical well-being compared to other generations [6]. Gen Z and millennials both experience high levels of mental health and sleep concerns [7-9]. Over half (57%) of Gen Z, and nearly half of millennials (46%) report experiencing depressive symptoms and anxiety [9], and these generations have the highest average self-reported stress compared to other generations [8]. Gen Z and millennials’ average stress on a 10-point scale is 5.3 and 5.7, respectively, while other generations range from 3.3 to 5.1 [8]. In addition to mental health concerns, nearly 40% of Gen Z and millennials rate their sleep as “fair” or “poor” [10], and more than two-thirds of Gen Z and millennials use a sleep aid to help them fall asleep or stay asleep [7]. There is a need to address the growing mental health and sleep concerns in GenZennials.

### Addressing Mental Health and Sleep Through Spirituality

Spirituality may be a promising approach for improving mental health and sleep in GenZennials. Gen Z and millennials identify as spiritual and engage in spiritual practices: 77% of Gen Z consider themselves spiritual, and 51% of millennials report feeling a deep sense of spiritual peace and well-being at least once per week [11,12]. Additionally, more than two-thirds (64%) of adult GenZennials (ie, ages 18-35 years) in the US report consuming web-based content related to religious or spiritual beliefs, values, ideas, or practices [13]. Spirituality has been linked to positive mental health outcomes and improved sleep across adult or older adult populations [14,15]. In a review summarizing the evidence of the relationship between spirituality and mental health, depressive symptoms were the most frequently investigated mental health outcome in relation to spirituality. Higher levels of spirituality were consistently linked to lower depressive symptoms [15]. However, the relationship between spirituality and anxiety was inconsistent. Two studies reported a correlation between spirituality and lower anxiety while 3 studies found no significant relationship [15]. Similarly, in one study of university students (ie, ages 17-23 years) in Hong Kong, lower levels of spiritual well-being were associated with higher levels of depressive symptoms and anxiety [16], suggesting there may be a relationship between spirituality and depressive and anxiety symptoms in young adults. Despite these findings, no studies in the Lucchetti and colleagues [15] review focused on GenZennials, and most of the studies were dated (ie, published 1 to 2 decades ago). The relationship between spirituality and depressive and anxiety symptoms needs further examination, specifically among adult GenZennials.

In addition to depression and anxiety, spirituality may help GenZennials manage their stress. Spirituality has been associated with reduced stress among various populations, such as Iranian university students [17], Palestinian adults (ages 20 to 59 years old) [18], and among US adults during COVID-19 [19]. However, these studies included populations that ranged outside of GenZennials (ages 18 to 35 years) and were conducted in populations outside the United States. To date, there have been little to no studies on spirituality and its relationship to stress specifically among US GenZennials. Considering the desire for these generations to practice spirituality and the relationship of spirituality to depression and anxiety, studies are warranted to determine how spirituality is related to stress, informing ways that spirituality may serve as a stress management tool.

Along with poor mental health, GenZennials also report sleep disturbance. One of the major reasons GenZennials lose sleep is due to spending more time on their phones, especially on social media [7,20]. In fact, Gen Z report losing sleep sometimes (32%) or often (48%) due to social media use [7]. Spiritual practices have been shown to improve sleep among adults who have insomnia, but evidence on spirituality and sleep disturbance in GenZennials is currently lacking [14]. To our knowledge, there have been no studies that examine the relationship between spiritual self-care and sleep disturbance among adult GenZennials [14]. Further research is needed to explore the relationship between spirituality and sleep among adult GenZennials to inform approaches to reduce sleep disturbance in these generations.

Because GenZennials are digital natives, they are dominated by digital devices and in fact are more likely to use digital wellness apps as compared to digital mental health programs [6]. Out of 11,915 individuals who use digital wellness apps, 55% of Gen Z and 50% of millennials found these apps on their own [6]. Digital approaches may provide an accessible way to deliver spiritual health interventions for GenZennials for the management of mental and sleep-related health. A recent scoping review [21] revealed that research is sorely lacking on digital approaches addressing young adults’ spiritual and mental health in these populations. GenZennials report being open to receiving mental health services via digital apps with almost one-third of those with a mental health condition (32%) reporting using an app to treat or manage their mental health and nearly two-thirds of GenZennials (62%) reporting they would use an app for their mental health care [22]. Additionally, 20% of these generations also report using mobile apps to improve their sleep [23].

Sadly, the relationship between spirituality, mental health, and sleep in GenZennials remains poorly understood [7-9]. GenZennials report a connection to and involvement in spirituality and spiritual health practices that is unique from other generations [11,12]. This presents an opportunity to investigate how GenZennials view spirituality and spiritual health practices and their association with mental health (ie, depressive symptoms, anxiety, and stress) and sleep, as well as to develop digital spiritual-based interventions for GenZennials. Taken together, we were interested in addressing the following research questions: (1) What are the characteristics of GenZennials who use a spiritual self-care app? (2) What are GenZennial perceptions of a spiritual self-care app before and after use? (3) What is the relationship between user engagement in a spiritual self-care app and mental health, sleep, and spiritual well-being among GenZennials? Therefore, the purpose of this paper was to describe a sample of adult GenZennials who use a spiritual self-care app (ie, Skylight), describe how users engage with and perceive the app, and assess the relationship between the frequency of using the app with mental health, sleep, and spiritual well-being. Information from this study may be used to inform future digital interventions for GenZennials. We chose to use the Skylight app because, to the authors’ knowledge, it is one of the few nondenominational spiritual well-being apps that exists, is free, and was created with the aim to increase access to spiritual self-care for as many people as possible.

## Methods

### Ethics Approval

This study was a cross-sectional survey, approved by Solutions Institutional Review Board (protocol #2023/03/18). All participants of this study provided electronic consent prior to participating in the survey. Participants received a US $25 e-gift card as an incentive. To protect participant confidentiality, data were deidentified prior to analyses. Data sets generated during the study are available from the corresponding author upon request.

### Skylight App

Skylight is a free spiritual self-care app developed by the Radiant Foundation. Radiant Foundation’s mission is to create change at scale while positively impacting the day-to-day lives of individuals. Their goal with the Skylight app is to cultivate a more personal, positive place for faith and spirituality within individuals. Skylight features spiritual practices including music, yoga, prayer, mindful movement, intention setting, and meditation. While these practices can be done without a mobile app, digital apps meet GenZennials on the web, where they spend much of their time [3-5]. Skylight is accessible for free on the web or via mobile devices. First-time users are prompted to create a Skylight account using an email address to initiate an account.

### Participants

Skylight users were invited to participate in the study via their registered Skylight emails (~10,000 emails were sent) or through a link in the Skylight app. The link in the email or app led to a Typeform survey where respondents were first asked eligibility questions. Potential participants were 18 years or older, were registered Skylight users, and had used the app at least once. If potential participants were eligible, they were directed to a consent form. If ineligible, users were notified that they were ineligible to take the survey and encouraged to continue using the Skylight app.

### Survey

The cross-sectional survey was developed by 2 doctoral researchers. The survey was executed on Typeform and included questions related to demographics (eg, age, gender identity, and annual household income), frequency of using the app, user spiritual self-care and practice, user engagement and perceptions of the app, and 4 validated questionnaires to assess depressive symptoms and anxiety (Hospital Anxiety and Depression Scale [HADS]) [24], stress (Perceived Stress Scale 4 [PSS-4]) [25], sleep disturbance (Insomnia Severity Index [ISI]) [26], and spiritual health (Spiritual Health and Life-Orientation Measure General Version [SHALOM]) [27]. The frequency of using the Skylight app was determined with the following multiple-choice item: On average, how often do you use the Skylight app? (1) Less than once per week, (2) 1-3 times per week, (3) 4-5 times per week, and (4) more than 5 times per week. Responses were categorized into three groups: (1) low-frequency app use (<1 time per week), (2) moderate-frequency app use (1-3 times per week), and (3) high-frequency app use (4 or more times per week). User engagement and perceptions questions consisted of branching logic.

### Statistical Analysis

Analyses were conducted in Stata 15 (StataCorp LLC). Sample sizes varied across analyses due to not all questions being answered. Frequencies were calculated for all demographic variables and for questions related to spiritual self-care (Tables S1 and S2 in Multimedia Appendix 1), user engagement, and perceptions (Table S3 in Multimedia Appendix 1). Mean scores and SDs were calculated for mental health outcomes and sleep scores (Table S4 in Multimedia Appendix 1). Mean scores and SDs were also calculated for SHALOM, which includes values for what participants want their ideal spiritual well-being to look like and what their lived experience is for each spiritual domain (ie, personal, communal, environmental, and transcendental) [27]. Differences in mean scores (δ) were calculated to determine if there was spiritual dissonance (difference >1 means there is a lack of harmony between ideal and lived experience; Table S5 in Multimedia Appendix 1). Finally, linear regression models were used to assess user frequency and its relationship to mental health, sleep, and spiritual well-being outcomes, controlling for all demographic variables included in this study: age, gender identity, sexual orientation, race, annual household income, education level, and relationship status. In the linear regression models, moderate (yes=1, no=0) and high-frequency app use (yes=1, no=0) results are reported using low frequency as the reference group. Standardized betas, standard errors, confidence intervals, and *P* values are reported. Linear regressions were run for spiritual well-being by user frequency using the total mean score for the spiritual domain and overall spiritual well-being. Overall spiritual well-being was calculated by using the total mean of the lived experience across all domains. This is conceptually supported by [28], where total scores for each domain were calculated and the total lived scores for each domain were used in analyses. While [27] states that observing relationships for each domain separately provides more nuanced insight, we used the total lived experience score due to the overall consistency of scores across all domains.

## Results

### Overview

A total of 2967 eligible responses were collected. For this study, respondents were excluded from analyses if participants were older than 35 years and if the participants initiated the survey from sources outside the link provided in the email or app platform. The final sample included 475 participants.

Descriptive statistics for demographics on the final sample (N=475) can be found in Table 1. Overall, more than half (260/475, 54.7%) of the sample was Gen Z and nearly half (215/475, 45.3%) were young millennials, almost 54% (255/475) were female, and 68% (324/475) were White.

**Table 1:** Demographic characteristics of Skylight users (ages 18-35 years).

Demographics		Total, n (%)
**Age** **(years)** **(N=47** **5; mean 27.5, SD 5.2)**		
	Gen Z^a^ (18-28 years)	260 (54.7)
	Young millennial (29-35 years)	215 (45.3)
**Gender identity (N=475)**		
	Female^b^	255 (53.7)
	Male	196 (41.3)
	Transman	1 (0.2)
	Genderqueer	1 (0.2)
	Androgynous	1 (0.2)
	Multiple identities (includes intersex, nonbinary, 2-spirited, third gender, and agender)	19 (4)
	Not sure	2 (0.4)
**Sexual orientation (n=471)**		
	Asexual	14 (3)
	Bisexual	27 (5.7)
	Gay or lesbian	21 (4.5)
	Pansexual	12 (2.6)
	Queer	5 (1.1)
	Straight^b^	383 (81.3)
	Prefer not to say	9 (1.9)
**Race (N=475)**		
	American Indian or Alaska Native or Indigenous	13 (2.7)
	Asian or Asian American	16 (2.7)
	Black, African American, or Native African	80 (16.8)
	Native Caribbean or Afro-Caribbean Islander	1 (0.2)
	Native Hawaiian or Pacific Islander	12 (2.5)
	White, European American, Caucasian^b^	324 (68.2)
	Multiracial	20 (4.2)
	Prefer not to respond	6 (1.3)
	Other or no response	3 (0.6)
Hispanic or Latinx (n=474)		48 (1.1)
**Annual household income** **(US $) (n=474)**		
	Less than $49,999	121 (25.5)
	$50,000 to $74,999^b^	140 (29.5)
	$75,000 to $99,999	130 (27.4)
	$100,000 to $124,999	46 (9.7)
	$125,000 to $149,999	12 (2.5)
	$150,000 to $199,999	7 (1.5)
	More than $200,000	18 (3.8)
**Education level (n=472)**		
	Some or less than high school	16 (3.4)
	High school graduate	77 (16.3)
	Some college	104 (22)
	Associate’s degree	88 (18.6)
	Bachelor’s degree^b^	156 (33)
	Graduate degree	24 (5.1)
	Trade school	7 (1.5)
**Relationship status (n=474)**		
	Single	135 (28.5)
	Married^b^	206 (43.5)
	In a relationship	105 (22.1)
	Divorced, separated, or widowed	28 (5.9)

^a^Gen Z: Generation Z.

^b^Reference variable.

### Spiritual Self-Care and Practice

In total, 84% (399/475) of all users reported that they currently have a spiritual self-care practice. In total, 92% (437/475) of all users reported that spiritual well-being and self-care were important or very important to them (Table S1 in Multimedia Appendix 1). Table S2 in Multimedia Appendix 1 presents definitions of spiritual self-care and endorsement of the definitions by users who currently have a spiritual practice. In total, 36% (142/475) of users endorsed the definition of spiritual self-care as a practice that supports their connection to something greater than themself (ie, higher power, nature, and community). A quarter of the respondents endorsed the definition of spiritual self-care as a practice that helps them feel positive and energized, and 21% (82/475) endorsed the definition of spiritual self-care as a practice that supports their sense of meaning and purpose.

### User Engagement and Perceptions

Table 2 presents reasons users downloaded the Skylight app. Nearly 30% (130/475) of users downloaded the app for spiritual well-being, 26.3% (125/475) of users downloaded the app for overall health, and 12.5% (60/475) downloaded the app for mental health. Table S3 in Multimedia Appendix 1 presents user impressions of Skylight before and after using the app. When asked about their first impression of Skylight, 42.3% (200/475) of users thought Skylight was a spiritual wellness or self-care app and 17.1% (81/475) thought it was a mental health app. First impressions remained consistent with 61.2% (287/475) of Skylight users who, after using the app, said the app was a spiritual wellness or self-care app and 18.6% (87/475) who said it was a mental health app. Additionally, users were asked whether they felt that Skylight was accurately designed as a spiritual well-being or self-care app, which most (278/475, 58.7%) users felt that it was.

**Table 2:** Reasons users downloaded the Skylight app.

Download reasons	All users (N=475), n (%)
Spiritual well-being	130 (27.4)
Overall health	125 (26.3)
Mental health	60 (12.6)
Stress or anxiety	51 (10.7)
Recommended	32 (6.7)
Sleep	31 (6.5)
Self-care	27 (5.7)
Free	6 (1.3)
Try something new	4 (0.8)
Curious	5 (1.1)
Others	4 (0.8)

### Mental Health, Sleep, and Spiritual Well-Being Outcomes

Table 3 presents the means and SD for depression, anxiety, stress, and sleep among all Skylight users. On average, respondents in this study scored within the normal range (score of 0 to 7) for depression (mean score 6.9, SD 3.7) and approached borderline abnormal (score of 8 to 10) for anxiety (mean score 7.7, SD 5.1). Though there is no established cut-off for the PSS-4, a final score ≥6 indicates that individuals are experiencing high levels of stress based on population norms [29]. In our sample, respondents had slightly high levels of stress with an average score of 6.7 (SD 2.8). The average sleep disturbance score was 5.3 (SD 5.9).

**Table 3:** Self-reported mental health and sleep characteristics in Skylight users (N=475).

Mental health outcome	Mean (SD)
Depression (HADS^a^) (0 to 7=Normal)	6.9 (3.7)
Anxiety (HADS) (0 to 7=Normal)	7.7 (5.1)
Stress (PSS-4^b^) (0 to 6=Normal or Low stress)	6.7 (2.8)
Sleep (ISI^c^) (0 to 7=Not clinically significant insomnia)	5.3 (5.9)

^a^HADS: Hospital Anxiety and Depression Scale.

^b^PSS-4: Perceived Stress Scale 4.

^c^ISI: Insomnia Severity Index.

**Figure 1:**
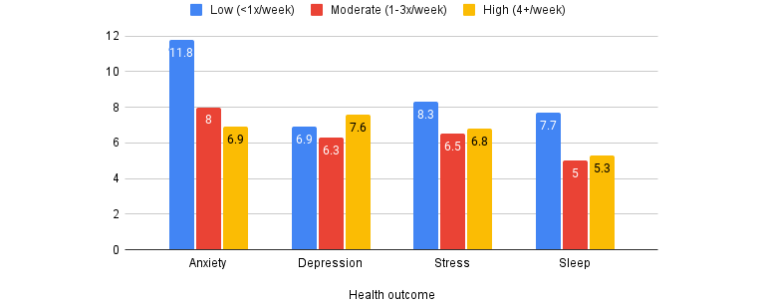
Mean mental health and sleep score outcomes by user frequency.

Figure 1 depicts mean scores calculated for each outcome by user frequency (Table S4 in Multimedia Appendix 1). We observed a general trend where moderate and high-frequency users had lower anxiety, stress, and sleep scores than low-frequency users. In contrast, low-frequency users had higher average depression scores than high-frequency users.

Overall results from the SHALOM survey revealed that users were generally spiritually well-aligned (Table S5 in Multimedia Appendix 1). In every domain for both ideal and lived experiences, users had an average score of 4.0 or higher on a 5-point scale, with the exception of the transcendental domain for lived experience (mean score 3.3, SD 0.5). The most notable difference between the average ideal and lived experience scores was in the transcendental domain (mean difference 0.9, SD 0.5). Given the consistent average scores across domains and among ideal and lived experiences, a total mean score was also calculated to capture the overall spiritual well-being of the study sample by calculating the averages for the ideal and lived experience using raw score data. The total difference was calculated by subtracting the total ideal and total lived experience means. The resulting score of –0.1 (SD 0.3) indicated that there is a negligible lack of harmony across all users in their overall spiritual health in which their lived experience matches closely to their ideal sense of spiritual wellness.

### Relationship Between Frequency of App Use and Mental Health, Sleep, and Spiritual Well-Being

Linear regression models were used to assess the association between the frequency of app use and each mental health, sleep, and spiritual well-being outcome, controlling for demographic variables. Table 4 presents nonsignificant relationships between app use and depression, stress, and sleep, respectively. However, app use was significantly associated with anxiety; compared to low frequency, moderate frequency (β=–2.01; *R*^2^=0.48; *F*_33,419_=11.76; *P*=.02), and high frequency use (β=–2.58; *R*^2^=0.48; *F*_33,419_=11.7; *P*=.004) had lower mean anxiety scores.

**Table 4:** Linear regression results of self-reported frequency of app use (predictor) and mental health and sleep (outcomes) in Skylight users.

Mental health	Depression (n=459)				Anxiety (n=452)				Stress (n=460)				Sleep (n=450)		
	β (SE)	95% CI	*P* value	β (SE)		95% CI	*P* value	β (SE)		95% CI	*P* value	β (SE)		95% CI	*P* value
Moderate app use^a^	.00 (0.75)	(–1.44 to 1.50)	.97	–.19 (0.86)		(–3.70 to 0.32)	.02	–.09 (0.51)		(–1.52 to 0.48)	.31	.01 (1.05)		(–1.91 to 2.24)	.88
High app use^a^	.16 (0.78)	(–0.32 to 2.76)	.12	–.25 (0.89)		(–4.34 to 0.83)	<.001	.00 (0.53)		(–1.02 to 1.06)	.97	.05 (1.09)		(–1.49 to 2.80)	.55

^a^Reference group: low-frequency app use.

Table 5 presents linear regression results for the lived experience of each spiritual well-being domain and for overall spiritual well-being lived experience by low, moderate, and high-frequency app use. No significant relationships were observed between app use and overall spiritual well-being. When examining the relationship across spiritual domains, app use was only significantly associated with the personal domain. Compared to low-frequency app use, moderate (β=–.28; *R*^2^=0.23; *F*_34,423_=3.73; *P*=.02) and high-frequency app use (β=–.47; *R*^2^=0.23; *F*_34,423_=3.73; *P*<.001) had lower mean scores in the personal domain lived experience.

**Table 5:** Linear regression results of self-reported frequency of app use (predictor) and spiritual well-being (outcomes) in Skylight users.

Mental health	Personal (n=447)			Communal (n=440)			Environmental (n=453)			Transcendental (n=446)			Overall spiritual well-being (n=429)		
	β (SE)	95% CI	*P* value	β (SE)	95% CI	*P* value	β (SE)	95% CI	*P* value	β (SE)	95% CI	*P* value	β (SE)	95% CI	*P* value
Moderate app use^a^	–.23 (0.12)	(–0.52 to –0.04)	.02	–.16 (0.12)	(–0.25 to 0.22)	.88	–.00 (0.16)	(–0.32 to 0.31)	.98	–.13 (0.10)	(–0.34 to 0.07)	.20	.05 (0.10)	(–0.16 to 0.26)	.63
High app use^a^	–38 (0.13)	(–0.71 to –0.22)	<.001	–.14 (0.12)	(–0.41 to 0.08)	.19	.13 (0.17)	(–0.31 to 0.35)	.90	–.14 (0.11)	(–0.36 to 0.07)	.19	–.03 (0.11)	(–0.24 to 0.18)	.78

^a^Reference group: low-frequency app use.

## Discussion

### Principal Findings

The purpose of this study was to describe the characteristics of adult GenZennial Skylight app users, observe how they engage with and perceive the app, and determine the relationship between frequency of use and mental health, sleep, and spiritual well-being outcomes. Skylight users report having a spiritual self-care practice and caring about their spiritual wellness. The number one reason they report downloading Skylight was for spiritual well-being. We found a significant relationship between the frequency of app use and anxiety. No significant relationships were found between the frequency of app use and depression, stress, or sleep. We also examined the relationship between the frequency of app use and spiritual well-being, where a significant relationship was found only within the personal domain of spiritual well-being.

### Skylight Users’ Spiritual Self-Care, Practice, and Perceptions

Most of the Skylight users had a spiritual self-care practice and reported that spiritual well-being and self-care were important or very important to them. More than half the users downloaded Skylight for reasons related to spiritual well-being and overall health. Even though GenZennials report being less religious than previous generations, the majority consider themselves to be spiritual or practice spirituality. Among 10,000 Gen Z, 77% reported themselves as spiritual [12,30]. Kinnaman [31] reported that 74% of Gen Z and 77% of millennials say that they would like to grow spiritually. Millennials are as likely as older generations to engage in spiritual practices, such as thinking about the meaning or purpose in life (55%), feeling or practicing weekly gratitude (76%), or feeling a deep sense of spiritual peace or well-being (51%) [11]. GenZennials may desire strategies to practice spiritual well-being that are not related to a specific religious practice, as Gen Z report resonating with parts and pieces of various spiritual traditions that conform to their values and needs [11-13]. It is important to note that Skylight is a spiritual self-care app that provides spiritual practices across faith traditions such as music, yoga, prayer, mindful movement, intention setting, and meditation. Spiritual self-care apps, such as Skylight, may offer an accessible way for GenZennials to practice spirituality that is amenable to their needs and provides variety based on their preferences and beliefs. More research is warranted to determine how these apps can be impactful to overall well-being.

In addition to the value that Skylight users had related to their spiritual self-care, 36% (142/475) also endorsed their spiritual self-care as “practices that support my connection to something greater than myself (ie, higher power, nature, and community).” In Springtide’s 2022 [12] survey of Gen Z, a 22-year-old adult explained how her spiritual practice helps her feel grounded and reminds her that there is more to life than immediate experiencing [30]. In a survey among 10,000 Gen Z, those who practiced spirituality or believed in a higher power described themselves to be flourishing and attributed their spiritual connection to their positive mental health [12,30]. Our findings further solidify the notion that GenZennials define and practice spirituality in ways that are unique from other generations. This is important information for the development and design of content for spiritual self-care apps that target GenZennials and can be used to develop spiritual self-care interventions in the future.

### Mental Health and Sleep Outcomes

Our regressions illustrated that more frequent use of the Skylight app was significantly associated with lower self-reported anxiety. This means that people who used the app more frequently reported less anxiety. There are few studies that have examined the relationship between spirituality and anxiety, many of the studies do not address Gen Z or young millennials and most are dated [32]. Engaging in spiritual self-care practices via a mobile app at least once per week may be a strategy for self-management of anxiety in GenZennials, but more studies are needed. Future research should not only continue to explore relationships but also determine the effects of an approach such as a spiritual self-care mobile app on anxiety in GenZennials.

Although in the descriptive statistics, the trends in mean scores for stress and sleep got better with the frequency of use, trends in depression scores did not get better. Our regressions illustrated that the frequency of using the Skylight app was not significantly associated with depression, stress, or sleep. This means that there was no relationship between how often Skylight users used the app and their self-reported depressive symptoms, stress, and sleep disturbance, respectively. This is likely because our sample did not have elevated symptoms; mean scores for both depressive symptoms and sleep were within the normal range and the mean stress score was just slightly above the threshold for high stress (score ≥6). While this study’s sample reported low mean scores for depression, stress, and sleep disturbance, it is important to note that the most reported reason for downloading Skylight was spiritual well-being. GenZennials seek self-care in various forms, such as exercise, life coaching, nutrition, astrology, and wellness apps [33,34]. GenZennials may not be thinking about their mental health “directly” and may be thinking about ways to feel better about life and themselves, thus seeking some form of spirituality in other ways. There is a need for more research that not only further examines the relationship of spiritual self-care to depressive symptoms, stress, and sleep but also examines the effect spiritual self-care delivered via a mobile app may have on these outcomes.

### Spiritual Well-Being

The total difference between the ideal and lived mean scores across all spiritual domains was –0.1 (SD 0.3), indicating that respondents are overall spiritually well-aligned. The difference between the ideal and lived experience scores in the transcendental domain was 0.9 (SD 0.5). The transcendental domain is defined as the relationship with, faith toward, or adoration of something or an entity beyond the human level (eg, spiritual force, cosmic force, transcendent reality) [35]. Fisher [35] defines a mean difference score of greater than 1 as “spiritual dissonance,” where the individual’s lived experience is not meeting the expectations or ideal sense of spiritual well-being within a domain. In this case, participants do not operationally fall into Fisher’s definition of spiritual dissonance, but there is more misalignment in their ideal and lived experiences within the transcendental domain compared to all other domains. This means that GenZennials’ lived experiences are not meeting up to their ideals related to connecting with a transcendental entity or spiritual force. This may be because over 30% of Gen Z identify as religiously unaffiliated [36]. GenZennials tend to disengage with organized religion or prescribe to one entity, but practice spirituality in other ways [12,30]. Fisher [35] suggests that for those who are nonreligious (or in this case, those who are spiritual and tend to not prescribe to organized religion), their transcendent lived experience score is likely to be lower as compared to those who are religious, who may have an entity they identify with and their feeling of connection to a transcendent is more natural and integrated into their practice. Gen Z’s approach to spiritual practice has been considered as “faith unbundled,” which means they combine elements such as beliefs, identity, practices, and community from a variety of sources that pertain to them and do not require a formal commitment [12]. Skylight’s content is built to apply across faith and preferences for spirituality practices. This includes (but is not limited to) intention setting, prayer, and discussions connecting with nature. Future studies should explore specific spiritual practices that help support GenZennials in feeling they have a relationship or are connected to something beyond themselves.

Linear regression results found that more frequent use of the app was associated with lower mean scores in the personal spiritual domain. The personal domain is defined as how an individual intrarelates to their self, such as their purpose, meaning, and values in life [35]. This means that people who use the app more frequently have lower scores (ie, score worse) in the personal domain. It could be that Skylight users with lower personal domain scores are accessing the app frequently because they are seeking ways to have a personal practice with themselves. Young adults have reported a need for autonomy in seeking health services and managing well-being, as it offers a way for users to have a personalized experience for themselves [37,38]. Finally, the content in the app is focused on the personal domain (eg, anxiety, stress, loneliness, and self-esteem). Further research is needed to determine how content can improve specific domains of spiritual well-being and how content can be better tailored for GenZennials.

### Strengths and Limitations

This study is novel and addresses an important gap in the literature by providing insights on the use of a spiritual self-care mobile app among GenZennials as a means to help them manage their mental health and sleep. However, the findings of this study should be interpreted considering its limitations. First, this was a cross-sectional survey and causal relationships cannot be determined from these analyses. Second, participant survey responses were self-reported, and thus, questions about participants’ frequency of using the Skylight app were categorical (eg, low, moderate, and high). Per Radiant Foundation’s request, IRB approval to use back-end data to obtain app use was not requested and respondent emails were only used to confirm that survey respondents were indeed Skylight users, to send incentives, and follow-up. Future studies should incorporate objective use data alongside self-reported data; for example, studies may use backend data such as duration (minutes) and number of sessions to determine the relationship between app use and outcomes. This would be particularly valuable in investigating questions related to how frequency, duration, and timeliness (eg, morning vs night) of app use is related to mental health and sleep outcomes. Third, there is potential bias resulting from participant self-selection, as all participants in this study were existing subscribers of the Skylight app. It is possible that individuals who use Skylight more frequently may have better mental health (eg, lower anxiety) to begin with; alternatively, individuals with elevated mental health symptoms may have opted not to participate due to burden [39]. To enhance the understanding of perceptions regarding spiritual well-being apps, future research should explore perceptions of spiritual well-being apps in those who do not already use them as well as across a range of demographic characteristics and in more diverse samples (eg, racial or ethnic minorities and trans or nonbinary individuals). It is worth noting that our sample primarily consisted of White, non-Hispanic, heterosexual, educated females, thus limiting the generalizability of our findings. The generalizability of our results may also be affected by the small sample size. To address this limitation, future studies should use stratified recruitment strategies to ensure a more diverse sample in terms of age, gender, income, race or ethnicity, and use patterns. See Table S6 in Multimedia Appendix 1 for a summary of suggestions for future research.

### Conclusions

This is the first study to describe the characteristics of GenZennials who use a spiritual self-care app (ie, Skylight) and examine how frequency of app use is associated with their mental health, sleep, and spiritual well-being. We found that most Skylight users downloaded the app for their spiritual well-being followed closely by their overall health and that using the app at least one time per week was associated with lower anxiety. In addition, while users were overall spiritually well-aligned, more frequent users had lower spiritual well-being scores in the personal domain of lived experience. Our findings inform the limited knowledge base surrounding digital spiritual self-care approaches toward addressing mental health and sleep concerns in GenZennials [21] and offer future directions to close these gaps. Future observational and intervention studies are necessary to further disentangle how the use of a spiritual self-care app may benefit mental health, sleep, and spiritual well-being over time.

## Appendix

Multimedia Appendix 1 Supplementary tables for the study.

## Abbreviations

Gen Z: Generation Z
GenZennials: Generation Z and young millennials
HADS: Hospital Anxiety and Depression Scale
ISI: Insomnia Severity Index
PSS-4: Perceived Stress Scale 4
SHALOM: Spiritual Health and Life-Orientation Measure General Version

## Data Availability

The data sets analyzed during this study are not publicly available, as this would violate Radiant Foundation and Skylight’s privacy policy. However, aggregated and anonymized data that are not associated with individual users and do not include personal information are available from the corresponding author upon reasonable request.
