# Supplementary material for: A Spiritual Self-Care Mobile App (Skylight) for Mental Health, Sleep, and Spiritual Well-Being Among Generation Z and Young Millennials: Cross-Sectional Survey
Source: JMIR Form Res. 2023 Sep 27;7:e50239. doi: 10.2196/50239 (PMC10568400; doi:10.2196/50239)
Supplement: Multimedia Appendix 1 [file formative_v7i1e50239_app1.docx]

**Table S1.** User spiritual well-being and self-care importance.

| Importance | All users (N=473), n (%) |
| --- | --- |
| Very important | 309 (65.3) |
| Important | 128 (27.1) |
| Neutral | 33 (6.9) |
| Less important | 3 (0.6) |
| Not at all important | 0 (0) |

**Table S2.** Endorsement of spiritual self-care definitions among users who have a spiritual self-care practice.

| Definition | All users (N=395), n (%) |
| --- | --- |
| Practices that support my connection to something greater than myself (ie, higher power, nature, and community) | 142 (35.9) |
| Practices that help me feel positive and energized | 98 (24.8) |
| Practices that support my sense of meaning and purpose | 82 (20.8) |
| Practices that help me feel calm and peaceful | 73 (18.5) |
| All of the above | 0 (0) |

**Table S3.** Users’ impressions of Skylight app purpose before and after use.

| App impression | Before-use (N=473), n (%) | After-use (N=469), n (%) |
| --- | --- | --- |
| Spiritual wellness or self-care | 200 (42.3) | 287 (61.2) |
| Mental health | 81 (17.1) | 87 (18.6) |
| Meditation | 69 (14.6) | 41 (8.7) |
| General health | 59 (12.5) | 0 (0) |
| Sleep | 22 (4.7) | 24 (5.1) |
| Prayer | 17 (3.6) | 20 (4.3) |
| Was not sure | 25 (5.3) | 10 (2.1) |

**Table S4.** Mean (SD) of mental health and sleep score outcomes by user frequency.

| Health outcome | Low (<1x/week) | Moderate (1-3x/week) | High (4+/week) |
| --- | --- | --- | --- |
| Anxiety | 11.8 (4.1) | 8.0 (5.1) | 6.9 (5.0) |
| Depression | 6.9 (4.2) | 6.3 (3.7) | 7.6 (3.6) |
| Stress | 8.3 (2.2) | 6.5 (2.8) | 6.8 (2.8) |
| Sleep | 7.7 (6.1) | 5.0 (5.9) | 5.3 (5.8) |

**Table S5.** Mean (SD) for spiritual health and life-orientation measure general version (SHALOM) domains in ideal and lived experience.

| Domain category | Ideal | Lived | Difference |
| --- | --- | --- | --- |
| Personal | 4.3 (0.5) | 4.2 (0.6) | 0.1 (0.5) |
| Communal | 4.3 (0.6) | 4.2 (0.6) | 0.1 (0.5) |
| Environmental | 4.1 (0.6) | 4.0 (0.8) | 0.1 (0.6) |
| Transcendental | 4.2 (0.6) | 3.3 (0.5) | 0.9 (0.5) |
| Overall | 4.0 (0.4) | 4.1 (0.5) | –0.1 (0.3) |

**Table S6.** Summary of future directions for research on generation Z (Gen Z) and young millennial spirituality.

| Evidence gap | Future study recommendations |
| --- | --- |
| Population (Gen Z and young millennials)^a^ | More studies on Gen Z and young millennials’ spirituality is warranted, and studies should aim to use diverse samples that reflect the population (eg, race or ethnicity and sexual orientation). |
|  | Relationships between spirituality and mental health and sleep outcomes should be explored among non-app users to compare with those who use the app. |
| Study design | Pilot intervention studies examining the feasibility or acceptability of a spiritual mobile app on mental health and sleep health outcomes are needed to inform full-scale clinical trials. |
|  | Pilot intervention studies examining the effects of spirituality on mental health and sleep outcomes are needed to understand the impact of spiritual self-care on said outcomes. |
|  | Consider sampling those who show elevated symptoms of depression, anxiety, stress, and/or sleep disturbance to examine the impact of spiritual self-care. |
|  | Examine relationships between specific spiritual practices (meditation, affirmations, stretching, and prayer) via a mobile app on mental health and sleep outcomes. |
|  | Mixed methods studies could provide further insights into what aspects of spirituality and spiritual practices Gen Z and young millennials practice and how these relate to their mental health and sleep. |
| Outcome measures | Consider specific measures that are more accurately designed to capture Gen Z and young millennials’ spiritual well-being. |
|  | Measures that include overall health, such as physical health, mental health, social connectedness (ie, belongingness to the social world), loneliness (ie, relationships with others), and life satisfaction, should be included in research examining Gen Z and young millennials’ spirituality. |
| Data and analysis | When possible, back-end user data should be used to gather objective data on app usage (eg, minutes per day, type of content viewed, and sessions completed). |
|  | Consider an analysis that determines the optimal “dose” (eg, time spent on the app and number of sessions) to see changes in depression, anxiety, stress, and/or sleep. |

^a^Gen Z: generation Z.
